# Self-triggered Asynchronous Optical Sampling Terahertz Spectroscopy using a Bidirectional Mode-locked Fiber Laser

**DOI:** 10.1038/s41598-018-33152-0

**Published:** 2018-10-04

**Authors:** R. Dawson Baker, N. Tolga Yardimci, Yi-Hsin Ou, Khanh Kieu, Mona Jarrahi

**Affiliations:** 1 University of Arizona, College of Optical Sciences, Tucson, 85721-0094 USA; 2 University of California Los Angeles, Electrical Engineering Department, Los Angeles, 90095 USA

## Abstract

We report a self-triggered asynchronous optical sampling terahertz spectroscopy system based on a single bidirectional mode-locked fiber laser and plasmonics-enhanced photoconductive nanoantennas. The fiber laser generates two optical mutually coherent pulse trains with a stable repetition rate difference, enabling time-domain terahertz spectroscopy without using any mechanical delay line, stabilization electronics, or external trigger. The resolved terahertz spectra over a 0.1–2 THz frequency range and a 30-second measurement time show more than a 70-dB dynamic range, revealing water absorption lines matching the HITRAN database, through a light-weight and compact spectroscopy setup.

## Introduction

Time-domain terahertz spectroscopy (THz-TDS) has diverse applications in material characterization, chemical detection, and industrial quality control^1–13^. However, most experimental implementations make deployment of the technology outside the laboratory environment difficult due to complex measurement requirements.

THz-TDS systems consist of a pulsed terahertz source and detector triggered by a femtosecond optical pump and probe beam, respectively. Generated terahertz pulses are incident on the sample to be analyzed and the transmitted or reflected terahertz pulses, which carry the sample’s spectral signature, are detected. A variable time-delay between the pump and probe beams allows temporal scanning of the generated terahertz pulses after interaction with the analyzed sample. The first THz-TDS systems used mechanical delay lines to scan across the temporal width of terahertz pulses, obtaining timing precision using quality optomechanics. The use of such delay lines however constrained THz-TDS designs in terms of acquisition speed, frequency resolution, and overall size of the measurement apparatus. These limitations were also found in optical pump-probe experiments in which scanning delay lines were used.

A promising alternative for introducing a variable time-delay between the pump and probe beams is optical sampling by cavity tuning (OSCAT)^14^, which controls the time-delay by tuning the repetition rate of the femtosecond laser. Although high spectroscopy speeds can be achieved using this approach, the tuning range constraint of the femtosecond laser repetition rate limits the frequency resolution of the spectroscopy system. In addition, the scanning time window is predefined when using this approach and an additional optical delay stage should be added to the system for an initial calibration. Another alternative technique for achieving high spectroscopy speeds is electronically-controlled optical sampling (ECOPS)^15^, which uses two femtosecond lasers. The difference between the repetition rates of these lasers is controlled electronically, usually by a piezoelectric transducer tuning the cavity length of one of the lasers. However, similar to OSCAT systems, an additional optical delay stage is necessary for an initial system calibration and adjustment of the scanning time window. Moreover, the use of two femtosecond lasers makes the system less compact than the traditional THz-TDS systems. High-speed THz-TDS using a single laser was recently demonstrated through polarization-controlled optical sampling (SLAPCOPS)^16^. The system uses a unique laser consisting of two ring resonators sharing the same pump diode and gain section. However, similar to OSCAT and ECOPS techniques, SLAPCOPS THz-TDS requires a phase control unit to precisely control the repetition rate difference between the pump and probe laser pulse trains and the jitter in the phase-control electronics can have detrimental effects on the spectral accuracy, dynamic range, and bandwidth of the spectroscopy system.

Another promising alternative for high-speed THz-TDS is asynchronous optical sampling (ASOPS). ASOPS was first demonstrated in the context of optical pump-probe experiments to address the limitations of mechanical delay lines^17–19^. Later, it was proved to have similar utility in THz-TDS and terahertz asynchronous optical sampling systems (ASOPS-THz-TDS) based on electronic delay lines or multiple lasers were developed over the last few years^20–23^. ASOPS-based terahertz dual-comb spectroscopy systems were also developed, which provide frequency resolutions finer than those of ASOPS-THz-TDS systems^24,25^. These ASOPS-based terahertz spectroscopy systems also eliminate mechanical delay lines and speed up data acquisition considerably. However, mechanical complexity is traded for electronic complexity, often requiring numerous phase-locked loops or stabilization electronics. Moreover, these systems often utilize two femtosecond lasers, which increase the complexity, size, and cost of THz-TDS systems.

A number of multiplexed mode-locked laser (MML) platforms have emerged recently, which are capable of emitting the pump and probe pulses from a single cavity simultaneously. An improved timing performance can be achieved by MMLs in free-running mode since environmental effects such as vibration and temperature variations tend to affect both pump and probe repetition rates similarly. MMLs may be multiplexed in wavelength, polarization, or propagation direction^16,26–40^. In some of these systems, the difference in repetition rates is stable enough to support ASOPS-THz-TDS and dual THz comb heterodyning^30,37^. For all MML platforms, pulse repetition rate differences are determined predominantly by linear effects of group velocity dispersion and birefringence. The effect of dispersion and birefringence on group delay, τ, and repetition rate difference, ${\rm{\Delta }}{f}_{r}$, may be summarized:

1 $${\rm{\tau }}=\,\frac{{\rm{\Delta }}{f}_{r}}{{f}_{r}^{2}}={\rm{\Delta }}\lambda {D}_{net}+\frac{{\rm{\Delta }}{n}_{ave}l}{c}$$

where *D*_*net*_ (ps/nm) is the net cavity dispersion, ${\rm{\Delta }}\lambda$ is the difference in center wavelengths, ${\rm{\Delta }}{n}_{ave}$ is the average birefringence, and *l* is the cavity length. When there is no average birefringence between the two pulse trains and the oscillator is wavelength multiplexed with a filtering mechanism, as in the recent demonstrations of ASOPS-THz-TDS and dual THz comb heterodyning^30,37^, very small sampling steps may only be accomplished by cavity dispersion management since there is a practical limit to repetition rate. By contrast, bidirectional schemes and polarization multiplexed systems do not have this limitation since they do not constrain the difference in center wavelength, and polarization multiplexed systems have the additional ability to offset group dispersion delay with birefringence^29,40^.

In this report, we demonstrate a system for self-triggered ASOPS-THz-TDS using bidirectional operation of a single mode-locked fiber laser^26,27,41^ and the efficient terahertz power generation and detection of plasmonics-enhanced photoconductive nanoantennas^42–49^. In doing so we eliminate the mechanical and electronic complexity and perform THz-TDS over a 0.1–2 THz bandwidth with dynamic range of more than 70 dB, without using any moving parts and/or servo control electronics.

## Experimental Apparatus and Methods

### Bidirectional Laser System

**Figure 1:**
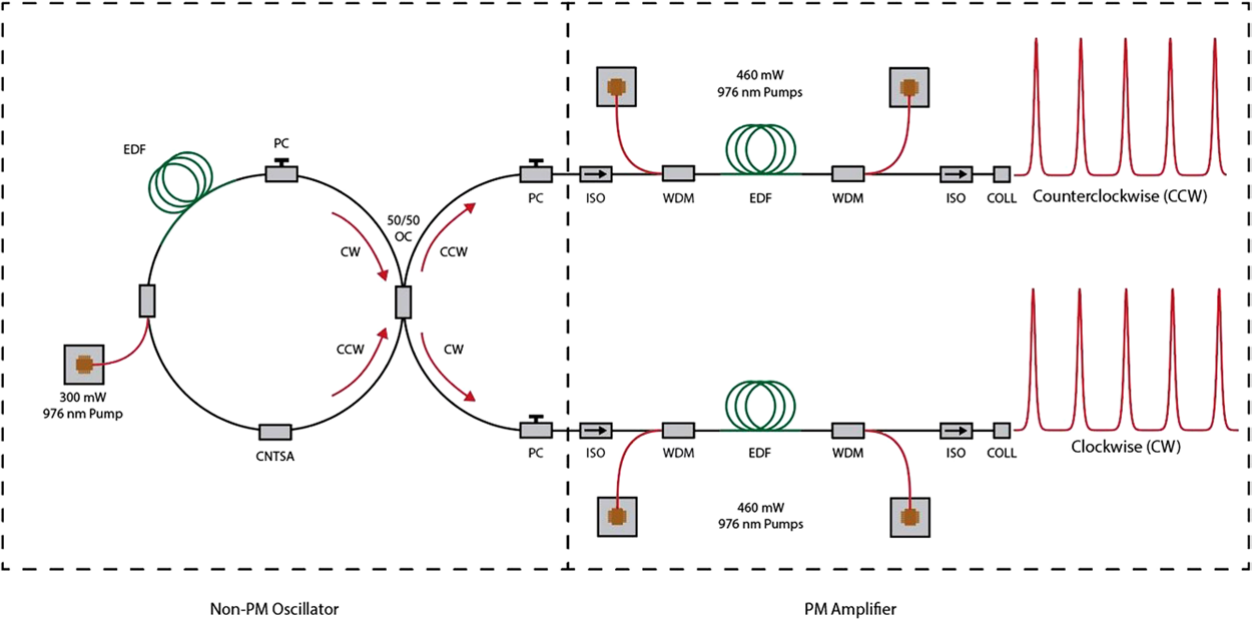
Schematic of the bidirectional mode-locked fiber laser. The master oscillator power amplifier system is comprised of a non-PM oscillator and PM erbium-doped fiber amplifiers (EDFAs).

**Figure 2:**
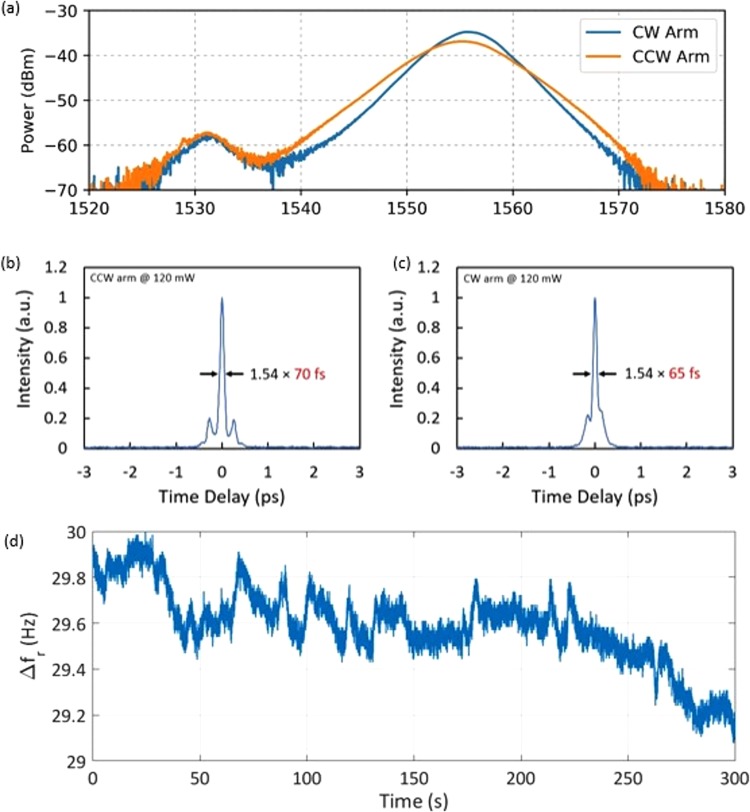
(**a**) Spectra of the CW and CCW laser outputs before amplification. The CW arm has a 6.0 nm bandwidth at a center wavelength of 1555.7 nm. Simultaneously, the CCW arm has a 7.7 nm bandwidth at a center wavelength of 1555.2 nm. (**b**,**c**) The amplified CCW and CW output pulses monitored using an autocorrelator, showing pulse compression down to 70 fs and 65 fs, respectively. (**d**) Measured repetition rate difference, ∆*f*_*r*_, over 300 seconds.

The laser system used in this experiment (shown in Fig. 1) is a modification of a design that has been used previously for free-running dual-comb spectroscopy in the near infrared regime^26,27^. The oscillator is a dispersion-managed soliton bidirectional oscillator with a net anomalous cavity dispersion^41^, which uses a carbon nanotube saturable absorber and requires tuning of a polarization controller to obtain bidirectional mode-locking. The oscillator has two outputs in the clockwise (CW) and counterclockwise (CCW) directions. Both oscillator outputs are made polarization maintaining (PM) to align the optical pump and probe fields reliably along the optimum axis of the plasmonics-enhanced photoconductive nanoantennas. These outputs are only 0.5 nm apart in center wavelength (Fig. 2(a)). After amplification in PM erbium-doped gain fibers, the pulses are compressed with a standard PM single-mode fiber (SMF) down to 70 fs in the CCW direction and 65 fs in the CW direction as can be seen in Fig. 2(b) and (c). Although mode-locking does not self-start, the system is capable of maintaining mode-locking for days at a time once started by adjusting the polarization in the laser cavity. The repetition rate *f*_*r*_ of the outputs is measured to be around 61.702 MHz, with a repetition rate difference ∆*f*_*r*_ of approximately 30 Hz, leading to a magnification factor of *M* = *f*_*r*_*/*∆*f*_*r*_ ≈ 2 × 10^6^ and a pulse-to-pulse delay of $\tau ={\rm{\Delta }}{f}_{r}/{f}_{r}^{2}\approx 8\,fs$.

The quality of the asynchronous optical sampling can be described purely in terms of the time stability of the magnification factor *M* = *f*_*r*_*/*∆*f*_*r*_, which is quantified as:

2 $$\frac{{\partial }_{t}M}{M}=\frac{{\partial }_{t}{f}_{r}}{{f}_{r}}-\frac{{\partial }_{t}{\rm{\Delta }}{f}_{r}}{{\rm{\Delta }}{f}_{r}}$$

Assuming that the repetition rate *f*_*r*_ and repetition rate difference ∆*f*_*r*_ fluctuations are (at worst) anti-correlated and their noises add:

3 $$\frac{{\sigma }_{M}}{M}\le \frac{{\sigma }_{{f}_{r}}}{{f}_{r}}+\frac{{\sigma }_{{\rm{\Delta }}{f}_{r}}}{{\rm{\Delta }}{f}_{r}}$$

The quantity *σ*_*M*_/*M*, which we term the ‘magnification deviation’, directly relates to the error in the asynchronous sampling rate, yielding non-uniform sampling. While each repetition rate *f*_*r*_ may have a deviation ${\sigma }_{{f}_{r}}$*/f*_*r*_ of 1 part in 10^7^–10^9^ over a single trace (typically 8–33 ms), for the repetition rate difference ∆*f*_*r*_ a comparable stability would require a ${\sigma }_{{\rm{\Delta }}{f}_{r}}\,$on the order of *µ*Hz or lower over the same duration^26^. Thus, it is clear that even though experiments have shown repetition rate differences to drift together in multiplexed single-oscillator systems like ours, the drift in ∆*f*_*r*_ is the dominant factor controlling the quality of the ASOPS-THz-TDS. In this experiment we observed a slow drift in ∆*f*_*r*_, which nevertheless allowed for averaging of 1000 traces over a ∼ 30 second interval to enhance the dynamic range of our ASOPS-THz-TDS.

### Time Domain Terahertz Spectroscopy System

**Figure 3:**
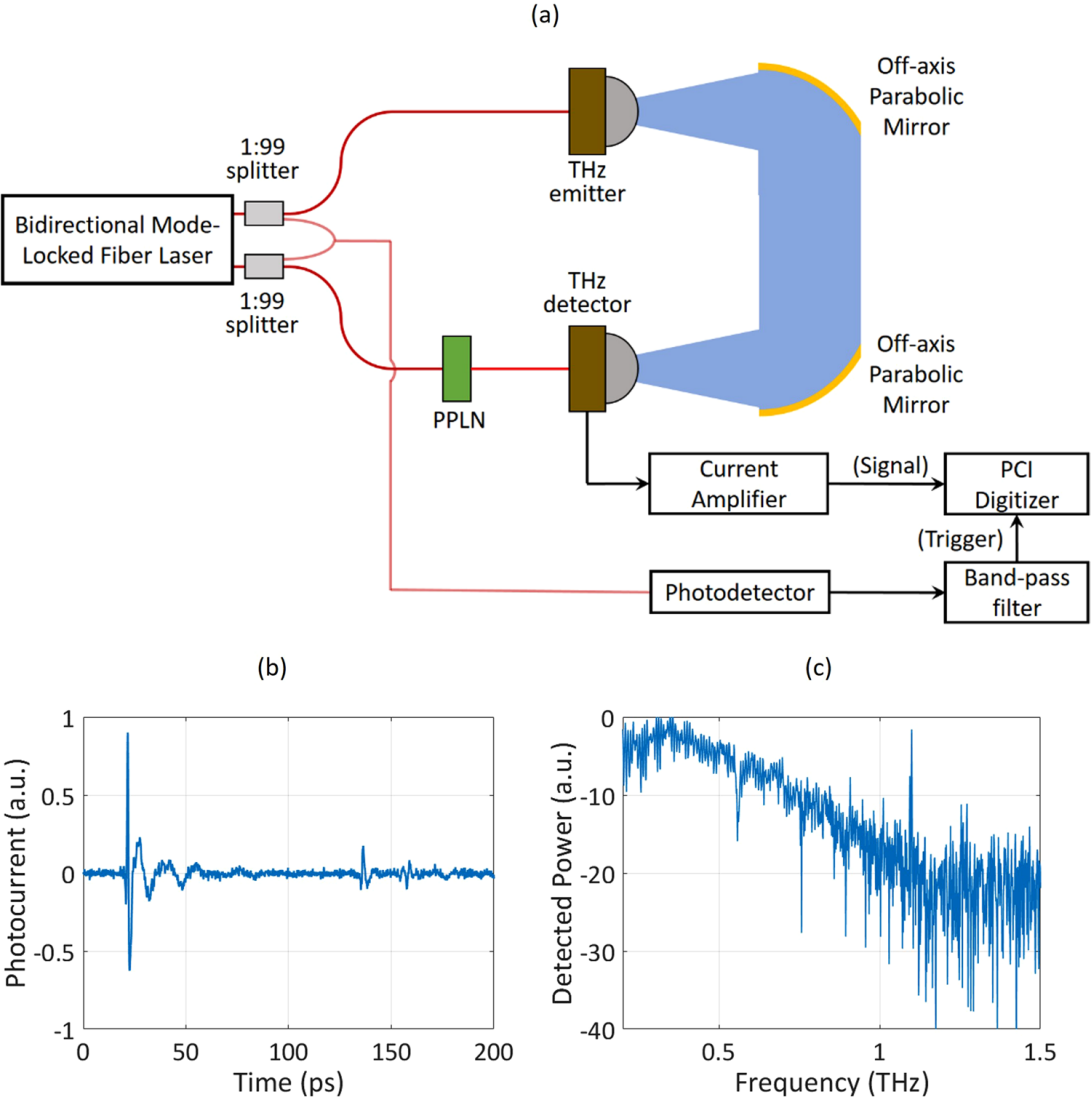
(**a**) Schematic of the THz-TDS setup with an external trigger. A 200-ps window of a single-trace time-domain photocurrent and the corresponding power spectrum obtained by the single-trace acquisition are shown in (**b**,**c**), respectively.

A schematic diagram of the THz-TDS setup is sketched in Fig. 3(a). Each arm of the bidirectional mode-locked fiber laser generates femtosecond pulses with a 120 mW power at ~1556 nm center wavelength. One laser output is focused onto a plasmonic photoconductive nanoantenna emitter array fabricated on an ErAs:InGaAs substrate to generate pulsed terahertz radiation^46^. The generated terahertz radiation is routed and focused onto a plasmonic photoconductive nanoantenna detector array^47^ fabricated on a low-temperature-grown GaAs substrate using two off-axis parabolic mirrors. The plasmonic photoconductive nanoantenna detector array is designed to operate at ~780 nm wavelength, so the other laser output passes through a periodically-poled Lithium Niobate (PPLN) crystal for second harmonic generation and the generated optical beam is focused onto the plasmonic photoconductive nanoantenna detector array. The generated photocurrent by the terahertz detector is amplified using a current amplifier (Femto DHPCA-100, amplification factor: 10^6^, bandwidth: 1.8 MHz) and recorded using a PCI digitizer card (AlazarTech ATS660). A trigger signal is typically required in ASOPS THz-TDS measurements to create an accurate time-base for the captured temporal traces. To generate this trigger signal, a small portion of the two laser output pulses is coupled and the interfered beam is sent to a photodetector (New Focus Model 1611). The photodetector output passes through a bandpass filter to obtain the generated interferogram from the two overlapping optical pulses. The generated interferogram, which serves as the trigger signal, is sent to the PCI digitizer for data acquisition and time-base creation.

## Results and Discussion

The acquisition time of a single photocurrent trace is inversely proportional to the repetition rate difference of the two laser outputs. The single-trace photocurrent acquisition time for the constructed THz-TDS system is 33 ms. Figure 3(b) shows a 200-ps window of the single photocurrent trace obtained from the terahertz detector. To obtain the power spectrum, the recorded photocurrent pulse is truncated to an 800-ps window with an asymmetric Hann window and the Fast Fourier Transform of the windowed pulse is calculated (Fig. 3(c)). The dynamic range of the resolved power spectrum from a single-trace acquisition is more than 20 dB, larger than that of the recently-demonstrated dual-comb spectroscopy systems based on dual-wavelength mode-locked lasers^30^. The spectral lines of water vapor at 0.557 THz and 0.753 THz are clearly observed in the resolved power spectrum.

**Figure 4:**
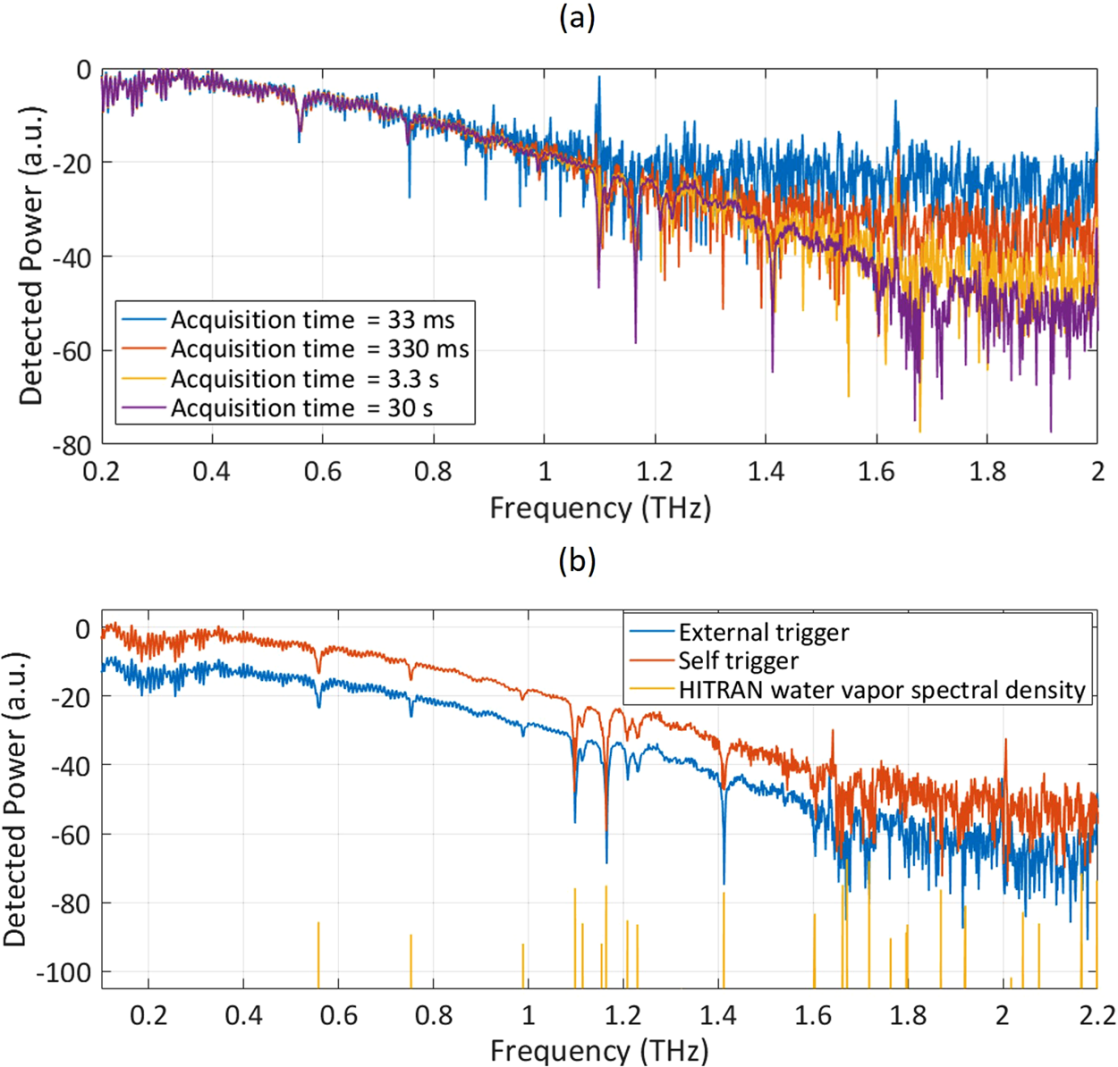
(**a**) The resolved power spectra for the acquisition times of 33 ms, 330 ms, 3.3 s, and 30 s. (**b**) The resolved power spectra over 30 seconds for externally-triggered and self-triggered systems in comparison with the HITRAN water absorption lines^54^.

In order to reduce the noise level, increase the bandwidth and dynamic range of the obtained terahertz spectrum, multiple photocurrent traces are recorded and averaged. Since the repetition rate difference, ∆*f*_*r*_, stays almost constant over a long time, no post-processing is required to fix the time-base of different captured photocurrent pulses. To demonstrate the effect of averaging multiple photocurrent traces, power spectra obtained over different acquisition times are compared in Fig. 4(a). As the number of the averaged traces increases, the noise level is reduced and a larger terahertz bandwidth is achieved. Approximately 1000 pulse traces are obtained in a 30-second data acquisition.

Next, the trigger system is eliminated and a self-triggered ASOPS THz-TDS system is constructed, which uses the generated photocurrent by the terahertz detector to trigger the digitizer card. The resolved power spectrum of the self-triggered THz-TDS system for a 30-second data acquisition time is shown in Fig. 4(b) (red curve). Comparison of the resolved power spectrum with the spectrum obtained using an external trigger (blue curve) shows that the self-triggered THz-TDS system offers the same signal-to-noise ratio, spectral bandwidth and accuracy as the externally-triggered THz-TDS system. This indicates the stability of the bidirectional mode-locked laser in terms of the repetition rate difference and the output power.

The ultimate factor that limits the frequency resolution of the ASOPS-THz-TDS system is the repetition rate of the laser outputs. However, the frequency resolution cannot usually reach this limit because of the uncertainties added due to the drift in the repetition rate difference of the two laser outputs. Additionally, the output photocurrent time-window duration used for calculating the power spectrum adds another limitation to the frequency resolution. The theoretical limit for the spectral resolution of the presented ASOPS THz-TDS system determined by the repetition rate of the laser outputs is 62 MHz. However, the use of an 800-ps time-window results in a 1.2 GHz spectral resolution. The spectral resolution can be further reduced to 62 MHz by using the full photocurrent temporal span, which naturally results in an increase in noise level and reduction in dynamic range. Moreover, sub-repetition-rate frequency resolution can be achieved by utilizing a dual-comb terahertz spectroscopy system through recording the terahertz pulse trains over long time-windows^25,50,51^ or interleaved sampling^52,53^, at the expense of a higher noise and reduced dynamic range.

To verify the accuracy of the resolved spectra, the frequency of the water spectral lines observed with the ASOPS THz-TDS system based on the bidirectional mode-locked laser is compared with the water absorption lines obtained from the HITRAN database^54^ (Fig. 4(b) yellow lines). As it can be seen in Fig. 4(b), a good agreement is found between the resolved water spectral lines by the ASOPS-THz-TDS system and the HITRAN dataset up to 2 THz. However, due to the increased noise level, it becomes harder to distinguish the spectral lines from the noise fluctuations above 1.65 THz. The mean deviation of the frequency of the spectral lines resolved by this system and those obtained from the HITRAN database is calculated as 563 MHz for frequencies below 1.65 THz, demonstrating the spectral accuracy of the spectroscopy system. It should be also noted that the environmental factors, such as humidity, temperature, and pressure are also partly responsible for this deviation.

Even though we show that there is no difference in terms of operation bandwidth and dynamic range between self-trigger and external-trigger operations, one should remember that the external trigger is necessary to have accurate knowledge of the timescale. Even though self-triggered operation is more than sufficient for various applications of terahertz science and technology, a time reference is still needed for many unique applications of THz-TDS where the temporal position of the measured signal is important.

Although we have demonstrated ASOPS-THz-TDS with a single free-running laser, reducing the need for a second laser, mechanical delay line, and locking electronics, the performance of the system can still be improved to offer higher dynamic ranges and broader bandwidths than the achieved 70 dB dynamic range over the 0.1–2 THz frequency range^23,55,56^. Several factors have limited the dynamic range and bandwidth of the demonstrated ASOPS THz-TDS system.

The main limitation on the system performance is the shape and duration of the optical pulses generated in the amplifiers using a soliton-compression scheme. Even though CW and CCW arms offer 65- and 75-fs FWHM pulses, respectively, their relatively large pulse pedestals broaden the overall pulse-width used for pumping/probing the terahertz source/detector, thus, ultimately producing slower transient photocurrent and narrower operation bandwidth for the ASOPS-THz-TDS system.

Deviations in the repetition rate difference of the two laser outputs, ∆*f*_*r*_, is another important factor impacting the most important specifications of the spectroscopy system. These deviations can cause two serious problems: aperiodic sampling with random fluctuations and time-base distortion. As the repetition rate difference drifts during data acquisition, the time difference between the two output pulses from the bidirectional laser varies uncontrollably, leading to random fluctuations in data sampling. An analogy can be made between this uncertainty in the repetition rate difference and the uncertainty in the position of mechanical delay stages used in traditional THz-TDS systems. A delay stage with low accuracy can cause such fluctuations in sampling as well, which are proven to limit the dynamic range of the spectroscopy system^57^. Similarly, the aperiodic sampling due to the deviations in the repetition rate difference of the two laser outputs is one of the main factors limiting the dynamic range of the presented spectroscopy system. The negative impact of the time-base distortion in the recorded output photocurrent traces is more apparent when averaging multiple traces. If the time-base of the traces are not the same, averaging can degrade the spectral resolution, add undesirable artifacts to the spectrum, and decrease the sharpness of the resolved spectral lines. Again, an analogy can be made between this time-base distortion and the time-base distortion caused by the repeatability of delay stage positions in traditional THz-TDS systems^58^.

Mode-locked oscillators with polarization control are typically sensitive to movement and environmental perturbations like temperature fluctuations and vibration. Thermal stabilization and vibration isolation can help to minimize the fluctuations in optical output power level and deviation of *M*. While phase-locking electronics may be complex and costly with two laser systems, single multiplexed oscillator ASOPS-THz-TDS might only require stabilizing one degree of freedom for high resolution performance, especially when using an external trigger.

Both the terahertz emitter and the detector have better performance under higher-power optical illumination^46,47^. Therefore, adding another amplification stage to the laser would help to increase the dynamic range and operation bandwidth of the ASOPS-THz-TDS system. Moreover, the terahertz detector used in this experiment is designed to operate at a 780-nm wavelength. Second harmonic generation is achieved with 40% conversion efficiency by using a PPLN crystal. The performance of the system would improve with a terahertz detector operating at the laser wavelength while offering similar performance.

Self-triggered ASOPS-THz-TDS has been demonstrated with a single bidirectional mode-locked fiber laser and plasmonics-enhanced photoconductive nanoantennas. Terahertz spectra over a 0.1–2 THz frequency range with more than a 70 dB dynamic range are resolved for a 30-second measurement time, revealing water absorption lines matching the HITRAN database, through a light-weight and compact spectroscopy setup.

## References

[1] Tonouchi Masayoshi (2007). Cutting-edge terahertz technology. Nature Photonics.

[2] Graham-Rowe Duncan (2007). Terahertz takes to the stage. Nature Photonics.

[3] Grischkowsky D., Keiding Søren, van Exter Martin, Fattinger Ch. (1990). Far-infrared time-domain spectroscopy with terahertz beams of dielectrics and semiconductors. Journal of the Optical Society of America B.

[4] Mittleman D.M., Jacobsen R.H., Neelamani R., Baraniuk R.G., Nuss M.C. (1998). Gas sensing using terahertz time-domain spectroscopy. Applied Physics B: Lasers and Optics.

[5] Kawase Kodo, Ogawa Yuichi, Watanabe Yuuki, Inoue Hiroyuki (2003). Non-destructive terahertz imaging of illicit drugs using spectral fingerprints. Optics Express.

[6] Woodward R.M., Wallace V.P., Arnone D.D., Linfield E.H., Pepper M. (2003). Journal of Biological Physics.

[7] Arnone Don, Ciesla Craig, Pepper Michael (2000). Terahertz imaging comes into view. Physics World.

[8] Van Zandt L. L., Saxena V. K. (1989). Millimeter-microwave spectrum of DNA: Six predictions for spectroscopy. Physical Review A.

[9] Federici John F, Schulkin Brian, Huang Feng, Gary Dale, Barat Robert, Oliveira Filipe, Zimdars David (2005). THz imaging and sensing for security applications—explosives, weapons and drugs. Semiconductor Science and Technology.

[10] Kemp, M. C. *et al*. Security applications of terahertz technology. In *Terahertz for Military and Security Applications*, vol. 5070, 44–53 (International Society for Optics and Photonics), 10.1117/12.500491.

[11] Nagel M, Först M, Kurz H (2006). THz biosensing devices: fundamentals and technology. Journal of Physics: Condensed Matter.

[12] Van der Weide D.W., Murakowski J., Keilmann F. (2000). Gas-absorption spectroscopy with electronic terahertz techniques. IEEE Transactions on Microwave Theory and Techniques.

[13] Nagai Naoto, Imai Tomoko, Fukasawa Ryoichi, Kato Koya, Yamauchi Koji (2004). Analysis of the intermolecular interaction of nanocomposites by THz spectroscopy. Applied Physics Letters.

[14] Wilk R, Hochrein T, Koch M, Mei M, Holzwarth R (2011). Terahertz spectrometer operation by laser repetition frequency tuning. JOSA B.

[15] Kim Y, Yee DS (2010). High-speed terahertz time-domain spectroscopy based on electronically controlled optical sampling. Optics letters.

[16] Kolano M, Gräf B, Weber S, Molter D, von Freymann G (2018). Single-laser polarization-controlled optical sampling system for THz-TDS. Optics Letters.

[17] Elzinga, P. A., Lytle, F. E., Jian, Y., King, G. B. & Laurendeau, N. M. Pump/probe spectroscopy by asynchronous optical sampling. **41**, 2–4. https://www.osapublishing.org/as/abstract.cfm?uri=as-41-1-2.

[18] Lytle, F. *et al*. Pump Probe Spectroscopy by Asynchronous Optical-Sampling. *Abstr*. *Pap*. *Am*. *Chem*. *Soc*. **193**, 47–ANYL, WOS:A1987G289600180 (1987).

[19] Kneisler, R. *et al*. Asynchronous Optical-Sampling - a New Combustion Diagnostic for High-Pressure Flames. *Abstr*. *Pap*. *Am*. *Chem*. *Soc*. **195**, 242–ANYL, WOS:A1988P599100547 (1988).

[20] Janke C., Först M., Nagel M., Kurz H., Bartels A. (2005). Asynchronous optical sampling for high-speed characterization of integrated resonant terahertz sensors. Optics Letters.

[21] Bartels A, Dekorsy T (2008). THz spectroscopy based on high-speed ASOPS. Tm-Technisches Messen.

[22] Holland, D. B. *Design*, *construction*, *and applications of a high-resolution terahertz time-domain spectrometer*, PhD, California Institute of Technology, http://resolver.caltech.edu/CaltechTHESIS:04222014-120506633 (2014).

[23] Klatt G (2009). Rapid-scanning terahertz precision spectrometer with more than 6 THz spectral coverage. Opt. express.

[24] Coddington I, Newbury N, Swann W (2016). Dual-comb spectroscopy. Optica.

[25] Yasui T, Kabetani Y, Saneyoshi E, Yokoyama S, Araki T (2006). Terahertz frequency comb by multifrequency-heterodyning photoconductive detection for high-accuracy, high-resolution terahertz spectroscopy. Appl. Phys. Lett..

[26] Mehravar S, Norwood RA, Peyghambarian N, Kieu K (2016). Real-time dual-comb spectroscopy with a free-running bidirectionally mode-locked fiber laser. Appl. Phys. Lett..

[27] Ou, Y. H. *et al*. Octave-spanning dual-comb spectroscopy with a free-running bidirectional mode-locked femtosecond fiber laser. In *Conference on Lasers and Electro-Optics*, *paper SM2L*.*3*, SM2L.3 (Optical Society of America, 2017), 10.1364/CLEOSI.2017.SM2L.3 (2017).

[28] Hu, G. *et al*. Multiwavelength, subpicosecond pulse generation from a SWNT-SA mode-locked ring birefringent fiber laser. in 2015 11th Conference on Lasers and Electro-Optics Pacific Rim (CLEO-PR) **2**, 1–2 (2015).

[29] Akosman AE, Sander MY (2017). Dual comb generation from a mode-locked fiber laser with orthogonally polarized interlaced pulses. Optics Express.

[30] Hu G (2018). Dual terahertz comb spectroscopy with a single free-running fibre laser. Scientific Reports.

[31] Zhao X, Li T, Liu Y, Li Q, Zheng Z (2018). Polarization-multiplexed, dual-comb all-fiber mode-locked laser. Photonics Research.

[32] Liu Y (2016). Unidirectional, dual-comb lasing under multiple pulse formation mechanisms in a passively mode-locked fiber ring laser. Optics Express.

[33] Zhao, X., Gong, Z., Liu, Y., Liu, J. & Zheng, Z. Coherent asynchronous sampling distance measurement using a single polarization-multiplexed ultrafast laser. in CLEO: 2014, paper STh4O.2 STh4O.2, 10.1364/CLEO_SI.2014.STh4O.2 (Optical Society of America, 2014) (2014).

[34] Zhao X, Zheng Z, Liu Y, Hu G, Liu J (2014). Dual-Wavelength, Bidirectional Single-Wall Carbon Nanotube Mode-Locked Fiber Laser. IEEE Photonics Technol. Lett..

[35] Zhao X (2016). Picometer-resolution dual-comb spectroscopy with a free-running fiber laser. Optics Express.

[36] Liao R (2018). Dual-comb spectroscopy with a single free-running thulium-doped fiber laser. Optics Express.

[37] Hu G (2017). Measurement of absolute frequency of continuous-wave terahertz radiation in real time using a free-running, dual-wavelength mode-locked, erbium-doped fibre laser. Scientific Reports.

[38] Zhao X (2011). Switchable, dual-wavelength passively mode-locked ultrafast fiber laser based on a single-wall carbon nanotube modelocker and intracavity loss tuning. Opt. Express, OE.

[39] Zhao X (2012). Fast, long-scan-range pump-probe measurement based on asynchronous sampling using a dual-wavelength mode-locked fiber laser. Opt. Express, OE.

[40] Krylov AA (2016). Generation regimes of bidirectional hybridly mode-locked ultrashort pulse erbium-doped all-fiber ring laser with a distributed polarizer. Appl. Opt., AO.

[41] Kieu Khanh, Mansuripur Masud (2007). All-fiber bidirectional passively mode-locked ring laser. Optics Letters.

[42] Jarrahi M (2015). Advanced Photoconductive Terahertz Optoelectronics Based on Nano-Antennas and Nano-Plasmonic Light Concentrators. IEEE Transactions on Terahertz Sci. Technol..

[43] Berry CW, Wang N, Hashemi MR, Unlu M, Jarrahi M (2013). Significant performance enhancement in photoconductive terahertz optoelectronics by incorporating plasmonic contact electrodes. Nat. Commun..

[44] Yang SH, Hashemi MR, Berry CW, Jarrahi M (2014). 7.5% Optical-to-Terahertz Conversion Efficiency Offered by Photoconductive Emitters With Three-Dimensional Plasmonic Contact Electrodes. IEEE Transactions on Terahertz Sci. Technol..

[45] Yardimci NT, Yang SH, Berry CW, Jarrahi M (2015). High-Power Terahertz Generation Using Large-Area Plasmonic Photoconductive Emitters. IEEE Transactions on Terahertz Sci. Technol..

[46] Yardimci NT, Lu H, Jarrahi M (2016). High power telecommunication-compatible photoconductive terahertz emitters based on plasmonic nano-antenna arrays. Appl. Phys. Lett..

[47] Yardimci NT, Jarrahi M (2017). High Sensitivity Terahertz Detection through Large-Area Plasmonic Nano-Antenna Arrays. Sci. Reports.

[48] Li X, Yardimci NT, Jarrahi M (2017). A polarization-insensitive plasmonic photoconductive terahertz emitter. AIP Adv..

[49] Yardimci NT, Cakmakyapan S, Hemmati S, Jarrahi M (2017). A High-Power Broadband Terahertz Source Enabled by Three-Dimensional Light Confinement in a Plasmonic Nanocavity. Sci. Reports.

[50] Hsieh YD (2013). Terahertz comb spectroscopy traceable to microwave frequency standard. IEEE Transactions on Terahertz Sci. and Technol..

[51] Finneran IA (2015). Decade-spanning high-precision terahertz frequency comb. Phys. Rev. Lett..

[52] Hsieh YD (2014). Spectrally interleaved, comb-mode-resolved spectroscopy using swept dual terahertz combs. Sci. Rep..

[53] Yasui T (2015). Super-resolution discrete Fourier transform spectroscopy beyond time-window size limitation using precisely periodic pulsed radiation. Optica.

[54] Gordon I.E., Rothman L.S., Hill C., Kochanov R.V., Tan Y., Bernath P.F., Birk M., Boudon V., Campargue A., Chance K.V., Drouin B.J., Flaud J.-M., Gamache R.R., Hodges J.T., Jacquemart D., Perevalov V.I., Perrin A., Shine K.P., Smith M.-A.H., Tennyson J., Toon G.C., Tran H., Tyuterev V.G., Barbe A., Császár A.G., Devi V.M., Furtenbacher T., Harrison J.J., Hartmann J.-M., Jolly A., Johnson T.J., Karman T., Kleiner I., Kyuberis A.A., Loos J., Lyulin O.M., Massie S.T., Mikhailenko S.N., Moazzen-Ahmadi N., Müller H.S.P., Naumenko O.V., Nikitin A.V., Polyansky O.L., Rey M., Rotger M., Sharpe S.W., Sung K., Starikova E., Tashkun S.A., Auwera J. Vander, Wagner G., Wilzewski J., Wcisło P., Yu S., Zak E.J. (2017). The HITRAN2016 molecular spectroscopic database. Journal of Quantitative Spectroscopy and Radiative Transfer.

[55] Vieweg N (2014). Terahertz-time domain spectrometer with 90 dB peak dynamic range. J Infrared Milli Terahz Waves.

[56] Nandi U, Norman JC, Gossard AC, Lu H, Preu S (2018). 1550-nm Driven ErAs:In(Al)GaAs Photoconductor-Based Terahertz Time Domain System with 6.5 THz Bandwidth. J Infrared Milli Terahz Waves.

[57] Jahn D (2016). On the influence of delay line uncertainty in THz time-domain spectroscopy. Journal of Infrared, Millimeter, and Terahertz Waves.

[58] Molter D, Trierweiler M, Ellrich F, Jonuscheit J, Von Freymann G (2017). Interferometry-aided terahertz time-domain spectroscopy. Optics express.

